# Erectile Dysfunction in Patients with Mental Disorders: Challenges in Diagnosis and Intervention from a Family Medicine Perspective

**DOI:** 10.1192/j.eurpsy.2025.2297

**Published:** 2025-08-26

**Authors:** C. G. Coronell, L. E. De Miguel, I. J. Contreras, O. Pascual, L. R. Bello, A. Ferrandez, P. Guerrero

**Affiliations:** 1 Family Practice Department, CAP Vallirana, Vallirana (Barcelona); 2 Psychiatry Department, Hospital Sagrat Cor, Martorell (Barcelona); 3 Nursery Department, CAP Vallirana, Vallirana (Barcelona); 4 Family Practice Department, CUAP La Solana, Sant Andreu de la Barca (Barcelona); 5 Family Practice Department, Centro de Salud Guadarrama, Madrid; 6 Family Practice Department, Centro de Salud de Nájera., La Rioja, Spain

## Abstract

**Introduction:**

In Spain, erectile dysfunction (ED) affects around 19% of men aged 25 to 70, with higher prevalence in older age groups. Family doctors are key for early ED detection, yet it is often underdiagnosed due to limited sexual health training and poor communication. Many patients hesitate to discuss sexual issues, and short consultation times worsen this problem. Early diagnosis is crucial for effective intervention, improving both quality of life and health outcomes.

**Objectives:**

To evaluate the effects of chronic diseases and medications on ED and identify commonly used treatments.

**Methods:**

This retrospective review focused on patients with late-diagnosed ED. We assessed the delay in reporting symptoms and contributing factors such as embarrassment and consultation constraints. The study also explored the impact of chronic conditions like diabetes and hypertension, and the role of psychotropic medications. Data were gathered from the Sexology Patient Database at Vallirana Primary Care Centre (CAP), covering 255 sexual dysfunction cases. The review aimed to understand diagnostic delays and the influence of chronic conditions and medications on ED.

**Results:**

Of 255 sexual dysfunction diagnoses, 193 were ED, making up 75.69%. The average patient age was about 62, with an average delay of 1.95 years before seeking help. ED comorbidities included Cardiovascular and Metabolic Diseases: 33.3%, Musculoskeletal and Articular Diseases: 31.25%, Psychiatric and Sleep Disorders: 29.2%, Digestive Diseases: 20.83%, Respiratory Diseases: 18.75%, Urogenital Disorders: 18.75%, Dermatological Disorders: 12.5%, Neurological Disorders: 10.42%, Endocrine Disorders: 8.33%, and Harmful Habits: 8.33%. Common medications were Antihypertensives: 16.7%, SSRIs: 14.6%, Proton Pump Inhibitors: 12.5%, Benzodiazepines: 10.4%, Antidiabetics: 10.4%, Statins and Fibrates: 10.4%, Bronchodilators and Inhaled Corticosteroids: 10.4%, Antiplatelets: 8.3%, and Other Medications.

**Conclusions:**

The high ED prevalence of 75.69% underscores major issues with timely diagnosis. The average 1.95-year delay highlights barriers to early treatment. Chronic conditions, especially cardiovascular and metabolic disorders, are closely linked to ED. The presence of psychiatric and sleep disorders indicates a complex interaction with sexual dysfunction. The variety of medications used, particularly antihypertensives and SSRIs, necessitates careful management to avoid adverse effects. Family doctors are crucial for diagnosing, managing ED, and referring to specialists. However, time limitations and lack of sexology training often result in underdiagnosis. Enhancing awareness can improve early detection and management, leading to better patient outcomes and timely specialist care.

**Disclosure of Interest:**

None Declared

